# CBD Versus CBDP: Comparing In Vitro Receptor-Binding Activities

**DOI:** 10.3390/ijms25147724

**Published:** 2024-07-15

**Authors:** Mehdi Haghdoost, Scott Young, Alisha K. Holloway, Matthew Roberts, Ivori Zvorsky, Marcel O. Bonn-Miller

**Affiliations:** 1 Nalu Bio Inc., 38 Keyes Avenue, Suite 117, San Francisco, CA 94129, USA; 2 Charlotte’s Web, 700 Tech Court, Louisville, CO 80027, USA; 3 Phylos Bioscience, 2455 NW Nicolai Street STE B-O6, Portland, OR 97210, USA

**Keywords:** agonist, antagonists, cannabidiol, cannabidiphorol, cannabinoid receptor

## Abstract

Phytocannabinoids with seven-carbon alkyl chains (phorols) have gained a lot of attention, as they are commonly believed to be more potent versions of typical cannabinoids with shorter alkyl chains. At the time of this article, cannabidiphorol (CBDP) and tetrahydrocannabiphorol (THCP) can both be purchased in the North American market, even though their biological activities are nearly unknown. To investigate their relative potency, we conducted in vitro receptor-binding experiments with CBDP (cannabinoid CB1/CB2 receptor antagonism, serotonin 5HT-1A agonism, dopamine D2S (short form) agonism, and mu-opioid negative allosteric modulation) and compared the observed activity with that of CBD. To our knowledge, this is the first publication to investigate CBDP’s receptor activity in vitro. A similar activity profile was observed for both CBD and CBDP, with the only notable difference at the CB2 receptor. Contrary to common expectations, CBD was found to be a slightly more potent CB2 antagonist than CBDP (*p* < 0.05). At the highest tested concentration, CBD demonstrated antagonist activity with a 33% maximum response of SR144528 (selective CB2 antagonist/inverse agonist). CBDP at the same concentration produced a weaker antagonist activity. A radioligand binding assay revealed that among cannabinoid and serotonin receptors, CB2 is likely the main biological target of CBDP. However, both CBD and CBDP were found to be significantly less potent than SR144528. The interaction of CBDP with the mu-opioid receptor (MOR) produced unexpected results. Although the cannabidiol family is considered to be a set of negative allosteric modulators (NAMs) of opioid receptors, we observed a significant increase in met-enkephalin-induced mu-opioid internalization when cells were incubated with 3 µM of CBDP and 1 µM met-enkephalin, a type of activity expected from positive allosteric modulators (PAMs). To provide a structural explanation for the observed PAM effect, we conducted molecular docking simulations. These simulations revealed the co-binding potential of CBDP (or CBD) and met-enkephalin to the MOR.

## 1. Introduction

**Figure 1:**
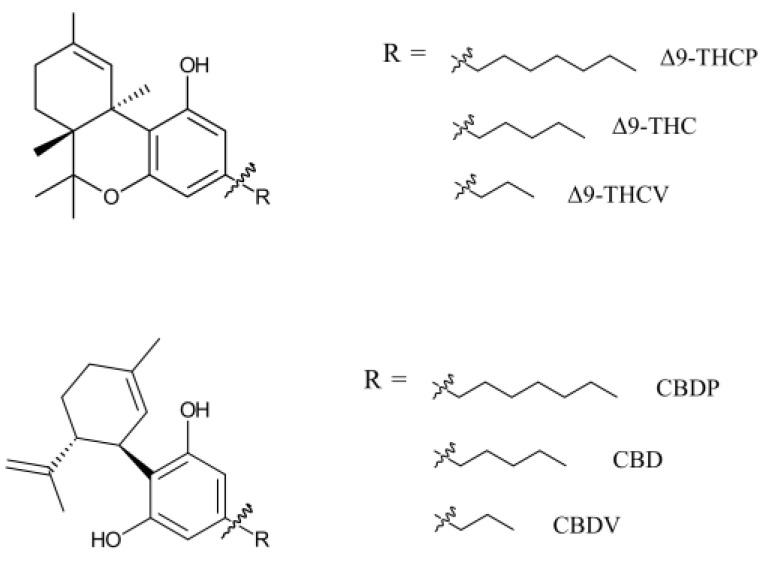
THC- and CBD-based phytocannabinoids with various alkyl chain sizes. Phytocannabinoids have multiple enantiomers/diastereomers. Only natural enantiomers are shown in this figure and used in this study.

Natural cannabinoids with seven-carbon alkyl chains (C7) have only recently been discovered in the cannabis/hemp plant. They are named with a phorol suffix. Cannabidiphorol (CBDP) and tetrahydrocannabiphorol (THCP) are the most discussed cannabinoids of this family (Figure 1). Research on synthetic THCP dates back to 1945, when Adams et al. reported cannabis-like activity for this compound [1]. Furthermore, progress in the synthesis of cannabinoids allowed the production of CBDP before its first detection in plant material [2]. THCP and CBDP were considered synthetic cannabinoids until 2019, when a trace amount of these cannabinoids was discovered in one cannabis variety [3], granting CBDP and THCP the title of natural phytocannabinoids. Compared to typical cannabinoids (five-carbon chain, C5) and varins (three-carbon chain, C3), phorols comprise a very small proportion of plant phytocannabinoids. In 2021, Bueno et al. found THCP in six *Cannabis sativa* L. chemotype extracts, but the content was only 0.002–0.014 *w*/*w*% of plant material [4]. The same plants contained >10% THC, demonstrating a THC/THCP ratio of higher than 1000. For the sake of comparison, a recent analytical study suggests that a significant portion of illicit cannabis in Canada contains 0.007 *w*/*w*% pesticides [5]. 

Information regarding the biological properties of phorols is scarce. An in vitro receptor-binding study suggests Δ9-THCP is a more potent CB1 agonist than Δ9-THC (Ki = 1.2 nM for Δ9-THCP and 40 nM for Δ9-THC), demonstrating a similar potency to that of the synthetic cannabinoid CP55,940 (Ki = 0.9 nM) [3]. The same study is also the only report on the in vivo activity of Δ9-THCP, showing that doses ranging from 2.5 to 10 mg/kg induce THC-like hypomotility, analgesia, and catalepsy in mice [3]. Although some individuals have shared their personal experiences with Δ9-THCP consumption in online forums, the human effects of Δ9-THCP have not been studied. The biological properties of CBDP are entirely unknown, and this phytocannabinoid has not been subject to any in vitro, in vivo, or human studies. However, reports on THCP being a more potent CB1 agonist than THC may promote the idea among the non-expert community that, similarly, CBDP might be more biologically potent than CBD. Further research on CBDP can shed more light on the biological effect of this phytocannabinoid.

Since their discovery in the cannabis plant, interest in phorol cannabinoids, especially THCP, has proliferated. Part of this interest is because of the intoxicating effect of THCP, which allows users to achieve a “legal high” by taking advantage of unintended allowances in the wording of the 2018 Farm Bill [6]. The definition of hemp in the 2018 Farm Bill as the plant *Cannabis sativa* L. with a ∆9-THC concentration not exceeding 0.3 percent by dry weight, created regulatory ambiguity, particularly for synthetic cannabinoids like THCP. This has led to the proliferation of THCP and CBDP products in the North American market, despite the concerning gap in knowledge about their biological properties. Indeed, CBDP products such as distillates, vapes, and gummies can be purchased in the United States (U.S.) through various e-commerce platforms. Some of these products contain more than 1 g of CBDP. Future cannabis and hemp plants may be capable of producing larger quantities of phorols through selective breeding and genetic modifications. However, at present, extracting CBDP from plant material is not financially viable due to its extremely low abundance in plant mass. As a result, CBDP is made using various organic synthesis methods [7]. As almost all synthesis routes lead to the formation of non-natural cannabinoid isomer impurities, the chemical synthesis of CBDP requires careful assessment of quality and purity. 

While products containing CBDP are emerging in the U.S. market, there has not been a single study documenting either the biological effects or toxicity of CBDP. To better understand the potential risks/benefits to consumer health and provide regulatory bodies with reliable scientific data for proper substance regulation, we believe understanding the biological effect of phorols should be addressed promptly. The aim of our study is to fill this critical knowledge gap by investigating the biological activity of CBDP. Specifically, we report its in vitro receptor-binding functionality and compare the data with that of CBD. By targeting receptors known to mediate the biological effects of CBD, we aim to determine whether similar or distinct mechanisms are at play for CBDP. This comparison is expected to elucidate the potential pharmacological profile of CBDP, thereby providing a scientific basis for its use. 

## 2. Results and Discussion

### 2.1. CB1 Antagonism

**Figure 2:**
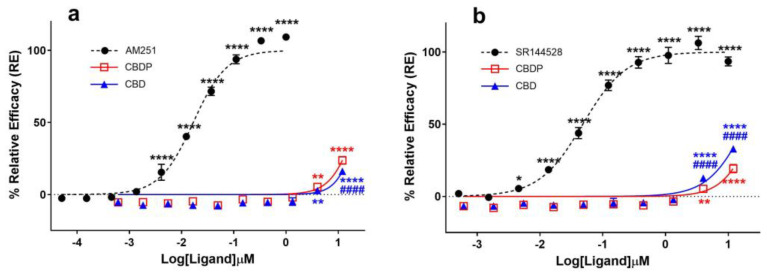
Comparative analysis of (**a**) CB1 and (**b**) CB2 antagonists (AM251, SR144528) and cannabinoids (CBD, CBDP) on cAMP activity following (±)-CP55,940 stimulation using a DiscoverX HitHunter cAMP XS+ assay. One hundred percent relative activity was normalized to maximum activity of AM251 (CB1) and SR144528 (CB2); 0% relative activity was normalized to response of vehicle control. **** *p* < 0.0001, ** *p* < 0.01, * *p* < 0.05 versus vehicle control. #### *p* < 0.0001 versus CBDP. Error bars represent the standard deviation of three independent measurements.

Several in vitro and in vivo studies have reported CBD as an antagonist of cannabinoid agonists at CB1 receptors [8,9,10], while some studies also characterize it as a potent negative allosteric modulator (NAM) of CB1 [11]. The antagonist properties of CBD and CBDP were assessed using the cAMP assay in the CB1-expressing CHO-K1 cell line by treating cells with different concentrations of CBD/CBDP, followed by CP55,940 agonist challenge (1 nM). We observed both compounds as similar weak antagonists (Figure 2a). For both CBD and CBDP, antagonist activity above the baseline appeared at 3 µM, and, at their highest tested dose (~12 µM), the activity reached ~20% the maximum response of AM251. Both compounds failed to reach IC_50_ values within the tested range of concentrations, while AM251 produced an IC_50_ = 6.1 nM, showing that the observed antagonist activity for CBD and CBDP is significantly weaker than that of potent CB1 antagonists. 

### 2.2. CB2 Antagonism

Reports on CBD acting as a potent antagonist of the CB2 receptor [10,12] prompted us to compare the ability of CBD and CBDP to antagonize the agonist effect of CP55,940 (1 nM) at this receptor using the cAMP assay, following the same protocol as that of CB1 antagonists. CBD demonstrated a moderately higher antagonist activity at the CB2 receptor compared to CB1 and also appeared to slightly outperform CBDP antagonism at this receptor (Figure 2b). At the highest tested concentration, CBD produced ~33% the maximum effect of SR144528, a potent and highly selective CB2 receptor antagonist/inverse agonist. At the same dose, CBDP showed a lower maximum response (~23%). This observed difference was small but statistically significant (*p* < 0.0001). While CBD is usually labeled as a potent CB2 antagonist [10], we observed CBD and CBDP as weak antagonists of the CB2 receptor, both failing to reach IC_50_ values within the tested concentrations (up to 12 µM). For comparison, an IC_50_ = 48.5 nM was calculated for SR144528.

### 2.3. 5HT-1A Agonism

**Figure 3:**
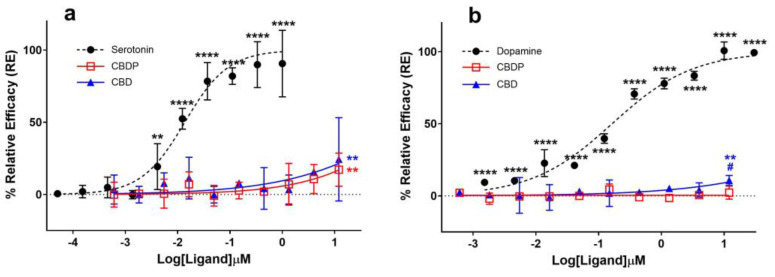
Agonist (**a**) 5HT-1A β-arrestin and (**b**) D2S cAMP assays for endogenous ligands (serotonin and dopamine) and CBD/CBDP after 90 min incubation time using PathHunter^®^ arrestin or DiscoverX HitHunter cAMP XS+ assays. One hundred percent relative activity was normalized to maximum stimulation of endogenous agonists and 0% relative activity to compound vehicle control. Error bars represent the standard deviation of three independent measurements. **** *p* < 0.0001, ** *p* < 0.01 versus vehicle control. # *p* < 0.05 versus CBDP.

It was traditionally believed that the anxiolytic properties of CBD [13] were related to its cannabinoid receptor activity. This view has been challenged as various new pharmacological properties of CBD have been discovered (for a review on this topic, see Nelson et al. [14]). In signal transduction studies, CBD acts as an agonist at the human 5HT-1A serotonin receptor [15]. Fogaça et al. (2014) reported that the effects of the intra-prelimbic prefrontal cortex injection of CBD on anxiety-like behavior in rats are modulated by 5HT-1A receptors [16]. Gregorio and colleagues have made a similar conclusion more recently [17]. Using a β-arrestin assay on a 5HT-1A-expressing CHO-K1 cell line, we observed comparable weak agonist properties for CBD and CBDP at the 5HT-1A receptor (Figure 3a). Both cannabinoids achieved ~20% the maximum response of serotonin, the endogenous ligand of the receptor, at the maximum tested dose (12 µM), without any significant difference between them. The data suggest an EC_50_ > 12 µM for CBD and CBDP, while under the same conditions, serotonin produced an EC_50_ = 0.02 µM, highlighting that the observed agonism for CBD and CBDP at 5HT-1A is very weak. 

**Figure 4:**
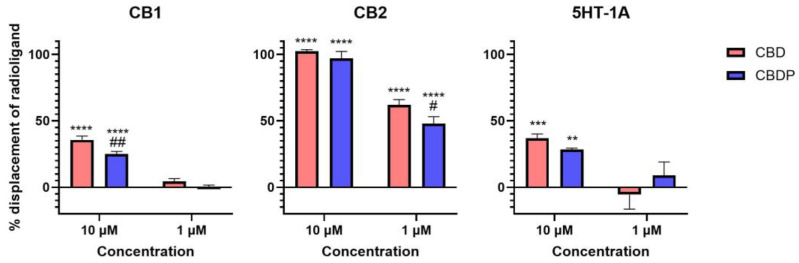
Displacement of radiolabeled [^3^H]-CP55940 (CB1 and CB2) and [^3^H]-8-Hydroxy-DPAT (5HT-1A) by cold CBD and CBDP at 1 and 10 µM. One hundred percent relative activity was normalized to maximum replacement by high control (5 µM cold CP55940 for CB1, 1 µM WIN55212-2 for CB2, and 0.3 µM serotonin for 5HT-1A), 0% relative activity to compound vehicle control. **** *p* < 0.0001, *** *p* < 0.001, ** *p* < 0.01 versus vehicle control. ## *p* < 0.01, # *p* < 0.05 versus CBD. Error bars represent the standard deviation of three independent measurements.

The activity curve of 5HT-1A agonism for CBD/CBDP suggests a more pronounced effect at lower concentrations compared to that for CB2 antagonism. However, the high data point variation observed in the 5HT-1A β-arrestin assay prevents us from making a reliable comparison. To facilitate a more comprehensive comparison, we assessed the binding affinity of CBD and CBDP to CB1, CB2, and 5HT-1A receptors at 1 and 10 µM, utilizing a radioligand binding assay (Figure 4). Both CBD and CBDP demonstrated the highest radioligand displacement at the CB2 receptor. At 1 µM, CBD and CBDP were able to replace approximately 50% of radiolabeled CP55940 at the CB2 binding site. Notably, CBD exhibited a slightly better affinity toward cannabinoid receptors compared to CBDP, which likely accounts for its marginally stronger antagonism at the CB2 receptor. The data suggest that among these three receptors, CB2 might be the main biological target of these two cannabinoids.

Although CBD and CBDP show activity at cannabinoid and serotonin receptors, our results suggest that their affinity is weak. However, CBD is commonly used in high doses in animals and humans to achieve high biological concentrations, enabled by its favorable safety profile [18]. For example, a systematic review of human clinical studies by Silmore et al. [19] shows that the C_max_ of CBD, after even a single high dose administration of CBD (1.5 g), can exceed 1 mg/mL (3.18 mM). These observations suggest that the doses and activity observed in this study are biologically relevant. 

### 2.4. D2 Agonism

Seeman (2016) reported that the antipsychotic effect of CBD [20] might be due to its interaction with the dopamine D2 receptor [21]. Cannabidiol was discovered to hinder the binding of radio-domperidone at dopamine D2 receptors, exhibiting a biphasic pattern similar to that of a dopamine partial agonist antipsychotic drug like aripiprazole [21]. In addition, the knockout of DOP-3, the dopamine D2-like receptor, eliminated the paralysis induced by CBD and CBDV (cannabidivarin) in the *C. elegans* model, suggesting the involvement of the dopamine receptor in the CBD biological effect [22]. We compared the agonist activity of CBD and CBDP at the dopamine D2 receptor using a cAMP assay in CHO-K1 Hunter cell lines; however, we did not observe notable agonist activity for either of the compounds (Figure 3b). CBD demonstrated a slight but statistically significant (*p* < 0.05) over-baseline activity at 12 µM, showing a 10.7% maximum response of dopamine (dopamine EC50 = 3.7 nM). CBDP did not show any sign of agonism, and it was unable to produce a significant signal within the tested concentration range.

It is noteworthy that the D2 dopamine receptor exists in two alternatively spliced isoforms, “long” and “short” (D2L and D2S), which have distinct brain distributions and functions [23]. Only the D2S isoform, which is believed to participate in presynaptic dopaminergic transmission [24], was used in this study. However, similar results would be expected for the D2L isoform, because agonists are reported to typically show similar IC_50_ values for the cloned human D2S and D2L receptors in vitro [25].

### 2.5. Mu-Opioid Antagonism/NAM

Two studies have identified CBD and its derivatives as negative allosteric modulators (NAMs) at the mu- (MOR) and delta-opioid (DOR) receptors [26,27]. Additionally, some synthetic CBD analogs have been recently shown to have potent NAM activity at the MOR by stabilizing the inactive conformation of the receptor. To compare the functionality of CBD and CBDP at the MOR, we first treated a MOR-expressing CHO-K1 cell line with 0.6 nM to 12 µM concentrations of CBD and CBDP for 30 min, followed by the addition of 1 µM of met-enkephalin (endogenous agonist, EC_50_ = 0.57 µM), and monitored the total internalization of the receptor. We selected the total internalization assay because the efficacy of opioid receptor ligands directly depends on their ability to induce receptor internalization [28].

**Figure 5:**
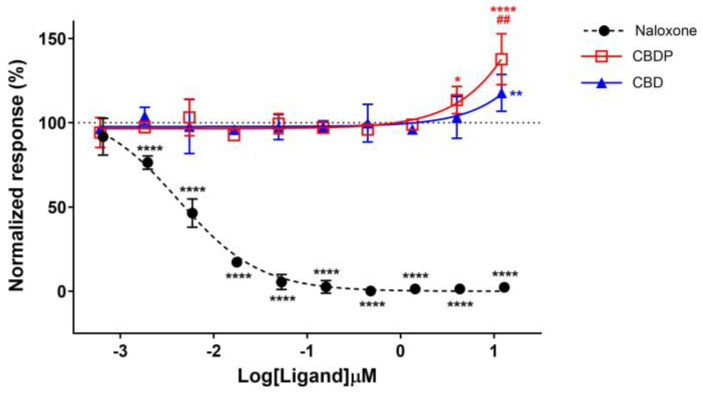
Normalized response in MOR total internalization assay for naloxone hydrochloride, CBD, and CBDP after 1 µM addition of met-enkephalin (endogenous agonist). One hundred percent relative activity was normalized to 1 µM met-enkephalin response and 0% relative activity to vehicle control. **** *p* < 0.0001, ** *p* < 0.01, * *p* < 0.05 versus vehicle control. ## *p* < 0.01 versus CBDP. Error bars represent standard deviation of three independent measurements.

According to previous reports, we were expecting a drop in receptor internalization for receptor antagonists or NAMs [29]. Indeed, naloxone hydrochloride, a strong antagonist of MOR, completely diminished the internalization response of met-enkephalin in a dose-dependent manner, producing an IC_50_ = 4.0 nM. However, an increase in the met-enkephalin internalization signal was observed when cells were pre-treated with high concentrations of CBD and CBDP (Figure 5). In particular, 10 µM CBDP led to a 37% increase in the met-enkephalin internalization signal, showing more activity than CBD at the same concentration (18% signal increase). These unexpected results are more aligned with the type of activity anticipated from positive allosteric modulators (PAMs) of the MOR. 

**Figure 6:**
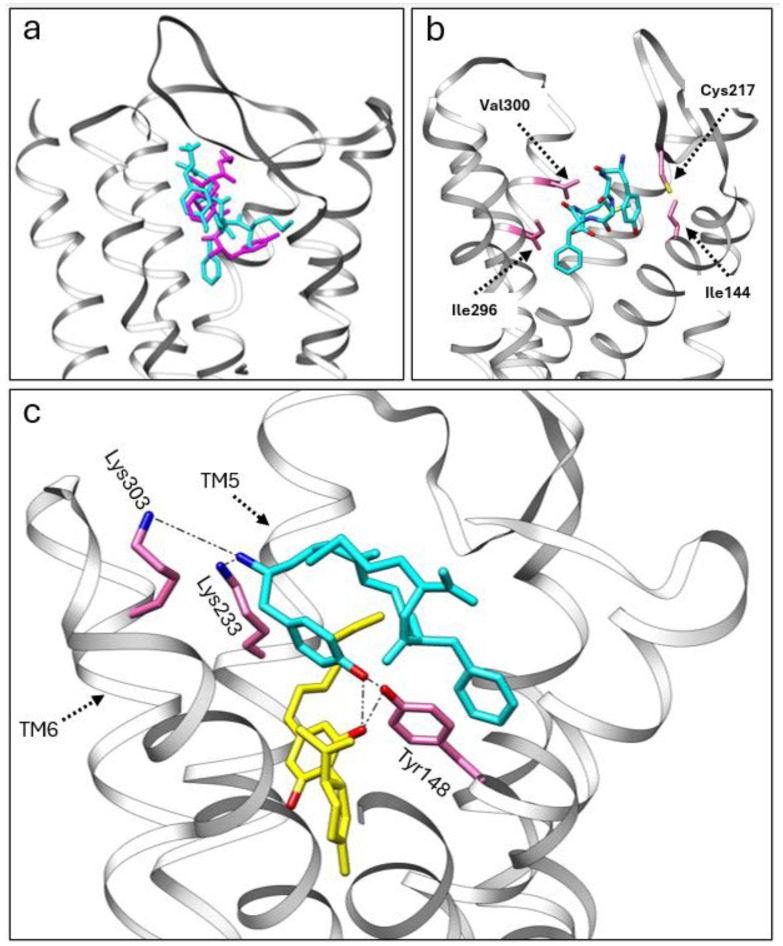
(**a**) A comparison of the crystal structure of DMAGO (magenta) with the calculated binding pose of met-enkephalin (cyan) inside the MOR pocket. (**b**) The calculated binding pose of met-enkephalin (cyan) within the MOR, highlighting specific amino acids (pink) involved in the interaction. (**c**) The co-binding of CBDP (yellow) and met-enkephalin (cyan) in the MOR orthosteric pocket, showcasing stable polar interactions with the receptor.

We utilized adocking simulation to gain insights into the potential allosteric modulation between CBD, CBDP, and met-enkephalin at the MOR. For this purpose, we used the crystal structure of the MOR with DAMGO (6DDF [30]) as the protein scaffold. Initially, we performed docking simulations of met-enkephalin alone, using Autodock Vina 1.2 [31]. Subsequently, we employed the multiligand-docking feature of Vina to investigate the co-binding interactions of CBD and CBDP with met-enkephalin at the receptor site. The top three docking poses for met-enkephalin (sequence: Tyr-Gly-Gly-Phe-Met) showed this peptide occupying a similar area of the receptor pocket as DAMGO (Figure 6a), likely due to the structural similarities between DAMGO and enkephalin. In the most energetically favorable binding pose (docking score = −11.167 kcal/mol), met-enkephalin sits in the middle of the binding pocket, with one end of this peptide interacting strongly with Cys217 and Ile144 and the other end with Ile296 and Val300 (Figure 6b).

Interestingly, the top three poses from the co-binding simulations of met-enkephalin and CBD/CBDP revealed that both the peptide and phytocannabinoid were situated within the orthosteric binding pocket of the receptor. In both the CBD and CBDP scenarios, the most energetically favorable pose featured a cannabinoid molecule positioned beneath the met-enkephalin, deep inside the MOR binding pocket (Figure 6c). This binding pose is stabilized by a hydrogen bond between one of the phenolic OH groups of the cannabinoid and Tyr148. Lipophilic amino acids Ile296, Leu219, and Leu232 are also involved in lipophilic interactions with the non-polar part of the cannabinoid molecules. The presence of cannabinoids shifts met-enkephalin to take a slightly different pose. The main difference is the extension of the N-terminus of met-enkephalin toward the top of TM5 and TM6 and the interaction with two polar amino acids, Lys233 and Lys303. Molecular dynamics calculations are required to understand how this change in binding mode can affect the conformational equilibrium of the receptor. However, we expect that these newly formed interactions will result in an outward push of TM5 and TM6 and lead to potentiating receptor activation by shifting the MOR equilibrium toward a fully activated state [32].

Comparing the docking score for the top poses of met-enkephalin (−11.167 kcal/mol), CBD + met-enkephalin (−14.263 kcal/mol), and CBDP + met-enkephalin (−14.413 kcal/mol) shows that co-binding is energetically more favorable than the binding of met-enkephalin alone. Besides newly formed interactions with the receptor, the proximity of CBD/CBDP to met-enkephalin in the receptor binding pocket allows interactions between CBD/CBDP and met-enkephalin, which contributes to the stability of the system. Among these intermolecular interactions, the hydrogen bonding between one of the OH groups of CBD/CBDP and the terminal tyrosine amino acid of met-enkephalin is of particular interest. The superior docking score for the co-binding of CBDP and met-enkephalin, compared to CBD + met-enkephalin, can account for the more pronounced allosteric effect of CBDP observed in vitro.

Comparing the allosteric effect of CBDP with that of BMS-986122, a known PAM of the MOR, can offer more insight into the extent of CBDP’s activity. In the stimulation of the guanosine triphosphate (GTP) turnover rate assay, BMS-986122 is reported to increase the activity of MOR to 166% at 10 µM concentrations [33]. In our total internalization assay, the same concentration of CBDP increased the receptor activity by 37%. Although different assays have been used for these two allosteric modulators, one may predict that CBDP is a weaker allosteric modulator than BMS-986122. However, our preliminary data suggest that the CBDP scaffold can be used to develop more potent allosteric modulators of opioid receptors. To validate the accuracy of our docking simulation, crystallographic or NMR binding studies should focus on the amino acid residues involved in the allosteric modulation between CBDP and met-enkephalin in the MOR. These studies could determine whether CBDP and BMS-986122 share similar or distinct allosteric binding sites and poses.

## 3. Materials and Methods

Naturally occurring enantiomers of CBD and CBDP ((−)-CBD and (−)-CBDP) were purchased as pure analytical standards (in acetonitrile) from Cayman Chemicals. (±)-CP55,940, AM251, SR144528, serotonin, dopamine, naloxone hydrochloride, and enkephalin were provided by Eurofins. 

In vitro assays were conducted by Eurofins Discovery (Fremont, CA, USA) using validated procedures and internal standard operation procedures (SOPs). A summary of the protocols is provided below:

### 3.1. PathHunter^®^ Arrestin Agonist Assay (Chemiluminescent Assay)

For the 5HT-1A agonist assay, PathHunter^®^ β-arrestin cells (CHO-K1) were expanded from freezer stocks according to standard procedures provided by Eurofins. The cell line manual was followed for cell growth (including cell culture media and supplementation, cell handling and preparation, etc.), test procedure, and signal detection. 

In summary, cells were seeded at a total volume of 20 µL (10,000 cells) into white-walled, 384-well microplates and incubated at 37 °C for the appropriate time prior to testing. After preparing stock acetonitrile solution at a 1 mg/mL concentration of ligand, compound intermediate concentrations were achieved by a 10-point series of 3-fold compound serial dilutions in a compound dilution buffer in a separate dilution plate. The concentration of each dilution was prepared at 5× of the final screening concentration. A total of 5 µL of sample solution was added to the cells (highest final concentration = 12 µM, highest final concentration of acetonitrile = 0.4%) and incubated at 37 °C for 90 min (5% CO_2_). Then, 12.5 μL Working Detection Solution was added, and the cells were incubated for 1 h at room temperature in the dark. The assay signal was generated through a single addition of 15 µL (50% *v*/*v*) of the PathHunter Detection reagent cocktail, followed by a one-hour incubation at room temperature. Microplates were read following signal generation with a PerkinElmer Envision instrument for chemiluminescent signal detection.

A more detailed assay protocol can be obtained from Eurofins (Catalog #: 93-0696C2) [34].

### 3.2. HitHunter cAMP XS+ Agonist Assay (Chemiluminescent Assay)

For the D2S agonist assay, cAMP CHO-K1 Hunter cell lines were expanded from freezer stocks according to standard procedures. The cell line manual was followed for cell growth (including cell culture media and supplementation, cell handling and preparation, etc.), test procedures, and signal detection.

In summary, cells were seeded in a total volume of 20 µL (10,000 cells) into white-walled, 384-well microplates and incubated at 37 °C for the appropriate time prior to testing. Before adding ligands, media were aspirated from the cells and replaced with 15 µL 2:1 HBSS (Hanks’ balanced salt solution)/10mM HEPES ((4-(2-hydroxyethyl)-1-piperazineethanesulfonic acid)/cAMP XS+ Ab reagent. After preparing a stock acetonitrile solution at a 1 mg/mL concentration of CBD and CBDP, compound intermediate concentrations were achieved by a 10-point series of 3-fold compound serial dilutions in a separate dilution plate, to generate a 4× sample in assay buffer containing 4× EC80 forskolin. A total of 5 µL of each sample solution was added to the cells (highest final concentration = 12 µM, highest final concentration of acetonitrile = 0.4%) and incubated at 37 °C for 30 min. The assay signal was generated through incubation with 20 μL cAMP XS+ ED/CL lysis cocktail for one hour, followed by incubation with 20 μL cAMP XS+ EA reagent for three hours at room temperature. Microplates were read following signal generation with a PerkinElmer Envision instrument for chemiluminescent signal detection.

A more detailed assay protocol is available free of charge on the Eurofins website (Catalog #: 495-0084C2) [35].

For the CB1 and CB2 antagonist assays, cells were pre-incubated with antagonists/compounds, followed by an agonist challenge at an ~EC_80_ concentration. Similar to the agonist assay, before adding the ligands, media were aspirated from cells and replaced with 15 µL 2:1 HBSS/10mM HEPES/cAMP XS+ Ab reagent. A total of 5 μL of 4× compound solution, prepared in a separate plate by a 10-point series of 3-fold compound serial dilutions in assay buffer (forskolin excluded), was added to the cells and incubated at 37 °C for 30 min. A total of 5 μL of the solution containing 4× EC80 of (±)-CP 55,940 and 4× EC80 of forskolin was added to the cells (final (±)-CP 55,940 concentration = 1 nM) and incubated at 37 °C for 30 min. The assay signal was generated through incubation with 20 μL cAMP XS+ ED/CL lysis cocktail for one hour, followed by incubation with 20 μL cAMP XS+ EA reagent for three hours at room temperature. A PerkinElmer Envision instrument was used for chemiluminescent signal detection.

More detailed assay protocols are available free of charge on the Eurofins website (Catalog #: 95-0071C2 for CB1 and 95-0183C2 for CB2 [35]).

### 3.3. PathHunter^®^ Total Internalization Antagonist/NAM Assay (Chemiluminescent Assay) 

For the MOR antagonist/NAM assay, PathHunter^®^ OPRM1 (opioid receptor mu 1) total GPCR (G protein-coupled receptors) internalization U2OS cell lines were expanded from freezer stocks according to standard procedures. The cell line manual was followed for cell growth (including cell culture media and supplementation, cell handling and preparation, etc.), test procedures, and signal detection.

In summary, cells were seeded in a total volume of 20 µL (10,000 cells) into white-walled, 384-well microplates and incubated at 37 °C overnight prior to testing. After preparing a stock acetonitrile solution at a 1 mg/mL concentration of ligand, compound intermediate concentrations were achieved by a 10-point series of 3-fold compound serial dilutions in a compound dilution buffer in a separate dilution plate. The concentration of each dilution was prepared at 10× of the final screening concentration. A total of 2.5 µL of the samples was added to the cells (highest final concentration = 12 µM, highest final concentration of acetonitrile = 0.4%) and incubated at 37 °C for 30 min. A total of 2.5 μL of enkephalin was added to the cells, to give a final enkephalin concentration of 1 µM, and the cells were incubated at 37 °C for 3 h. A total of 12 µL of detection reagent working solution was added, and the incubation was continued for 60 min at room temperature. A PerkinElmer Envision instrument was used for chemiluminescent signal detection.

A more detailed assay protocol is available free of charge on the Eurofins website (Catalog #: 93-0745C3) [36].

### 3.4. Radioligand Binding Assay (Radiometric Assay)

The compounds plate was first prepared by preparing 8 doses of reference (CP55940 for CB1, WIN55212-2 for CB2, and serotonin for 5HT-1A), starting from a 5mM DMSO stock solution by 5-fold serial dilutions. In addition, 10 mM and 1 mM DMSO stock solutions of CBD and CBDP were also prepared. A total of 750 nl of the reference and test compounds was transferred to a 96-well compound plate, and 150 µL assay buffer was added to each well to achieve a 5× final concentration. The plates were centrifuged for 30 s at 1000 rpm and then agitated at 600 rpm and room temperature for 5 min.

Separately, 50 µL of 0.5% *v*/*v* PEI was added to each well of UniFilter-96 GF/C plates. The plates were sealed and incubated at 4 °C for 3 h. After incubation, they were washed 2 times with ice-cold wash buffer.

The cell membrane was diluted with assay buffer, and 330 µL was transferred to 96 round deep-well plates to reach a concentration of 10 µg/well. A total of 110 µL of two concentrations of CBD and CBDP, as well as 8 concentrations of reference, was transferred from the compound plate to 96 round deep-well plates. The radiolabeled ligand was diluted in assay buffer, and 110 µL of this solution was transferred to 96 round deep-well plates to produce a 5× final concentration of radioligand (10 nM [^3^H]-CP55940 for CB1, 6.25 nM [^3^H]-CP55940 for CB2, and 1 nM [^3^H]-8-Hydroxy-DPAT for 5HT-1A). The plates were centrifuged at 1000 rpm for 30 s and then agitated at 600 rpm for 5 min (room temperature). The plates were sealed and incubated at 30 °C for 90 min. The incubation was stopped by vacuum filtration onto GF/C filter plates, followed by washing 4 times with ice-cold wash buffer. The plates were dried at 37 °C for 45 min. After the addition of 40 µL of scintillation cocktail, a Microbeta2 microplate counter was used to detect the radioactivity signals. 

### 3.5. Statical Analysis

Graph Pad version 10.2.2 was utilized for the data analysis in this study. Two-way ANOVAs with Tukey tests were used to compare the significance of differences between data points across groups. In terms of the data fitting for different types of assays, specific models were applied depending on the nature of the assay. A log(concentration) versus response curve with a variable slope was used to fit the data.

### 3.6. Docking Simulations (In Silico Studies)

The structural template for the MOR (6DDF) was obtained from the Protein Data Bank. Prior to the molecular docking analysis, parameters such as sidechain outliers and clash scores required refinement to ensure the most reliable results. The Chimera Dock Prep function was employed to prepare the receptors for docking simulations, which included the removal of solvent and water molecules. The incomplete sidechain was replaced with the Dunbrack 2010 rotamer library [37]. Hydrogen atoms were added, considering steric factors and H-bonds. Charges were assigned using the AMBER ff99bsc0 force field [38]. Once the receptor protein model was refined, we performed an energy minimization as a prerequisite for the molecular docking analysis. Autodock Vina [31] was utilized for the molecular docking analysis. The performance of three different scoring functions (Vina, vinardo [39], and a recently reported custom empirical set [40]) was assessed during the docking method validation. The scoring function that performed the best in the validation process was selected for the docking analysis. For the custom empirical set, the gauss1, gauss2, repulsion, hydrophobic, hydrogen bond, and rotation values were set at −0.049811, −0.007218, 0.756221, −0.031562, −0.469951, and 0.025722, respectively. 

To validate the docking method, the molecular docking was first conducted on a series of agonist/antagonist active and decoy ligands (450 ligands, with an active-to-decoy ratio of 1:10). The areas under the curve (AUCs) in the receiver operating characteristic (ROC) graphs were used to analyze the success of each model and scoring function [41]. Decoy ligands were obtained from the DUD.E database [42]. To prepare for the docking simulations, the 3D structure of the ligands was built from SMILES strings using Open Babel 3.1.1, and Gasteiger charges were assigned to each molecule.

## 4. Conclusions

The receptor-binding activities of CBD and CBDP are not dramatically different. In contrast to what has been observed for THC and THCP, the heptyl chain of CBDP does not improve the reported activities of CBD at the CB1, CB2, 5HT-1A, or D2S receptors. Instead, CBD appears to be a slightly more potent CB2 antagonist than CBDP. At concentrations higher than 3 µM, CBD showed activity at all the tested receptors, showcasing a complex therapeutic mechanism of action. However, the observed activities were weak (no IC_50_/EC_50_ was achieved), suggesting that a high dose of CBD is often required to achieve meaningful therapeutic benefits. CBDP demonstrated a surprising behavior at the MOR and, by increasing the total internalization of enkephalin, hinted toward acting as a PAM of this receptor. We used a molecular docking simulation to provide an explanation for the observed effect. 

We believe the data obtained provide an initial insight into the biological properties of CBDP and address a critical knowledge gap regarding its biological activity, through a comparison of its receptor-binding functionality to that of CBD. Data from this study suggest several avenues for future research on this rare phytocannabinoid. One area of future research should further investigate the potential role of CBDP as a PAM at the MOR, as this would suggest that CBDP could be effective in pain management. Further study using established PAMs as standards, and investigating other receptor signaling pathways (such as the cAMP pathway), is required to provide a better understanding of this unexpected activity. Future studies should also focus on comparing the activity of CBD and CBDP at other receptors that are typically reported to be targets of phytocannabinoids, such as GPR55, GPR119, and TRPV1.

## Data Availability

The original contributions presented in the study are included in the article, further inquiries can be directed to the corresponding author.
